# Rising Incidence of Early-Onset Liver Cancer and Intrahepatic Bile Duct Cancer: Analysis of the National Childhood Cancer Registry Database

**DOI:** 10.3390/cancers17071133

**Published:** 2025-03-28

**Authors:** Pojsakorn Danpanichkul, Yanfang Pang, Thanida Auttapracha, Omar Al Ta’ani, Thanathip Suenghataiphorn, Apichat Kaewdech, Mark D. Muthiah, Donghee Kim, Karn Wijarnpreecha, Amit G. Singal, Ju Dong Yang

**Affiliations:** 1 Department of Internal Medicine, Texas Tech University Health Sciences Center, Lubbock, TX 79409, USA; 2 Affiliated Hospital of Youjiang Medical University for Nationalities, Baise 533000, China; 3 National Immunological Laboratory of Traditional Chinese Medicine, Baise 533099, China; 4 Center for Medical Laboratory Science, Affiliated Hospital of Youjiang Medical University for Nationalities, Baise 533099, China; 5 Department of Microbiology, Faculty of Medicine, Chiang Mai University, Chiang Mai 50200, Thailand; 6 Faculty of Medicine, Chiang Mai University, Chiang Mai 50200, Thailand; 7 Department of Medicine, Allegheny Health Network, Pittsburgh, PA 15212, USA; 8 Department of Internal Medicine, Griffin Hospital, Derby, CT 06418, USA; 9 Gastroenterology and Hepatology Unit, Division of Internal Medicine, Faculty of Medicine, Prince of Songkla University, Songkhla 90110, Thailand; 10 Division of Gastroenterology and Hepatology, Department of Medicine, National University Health System, Singapore 119074, Singapore; 11 Division of Gastroenterology and Hepatology, Stanford University School of Medicine, Stanford, CA 94063, USA; 12 Division of Gastroenterology and Hepatology, Department of Medicine, University of Arizona College of Medicine, Phoenix, AZ 85004, USA; 13 Department of Internal Medicine, Banner University Medical Center, Phoenix, AZ 85004, USA; 14 BIO5 Institute, University of Arizona College of Medicine-Phoenix, Phoenix, AZ 85004, USA; 15 Division of Digestive and Liver Diseases, Department of Internal Medicine, University of Texas Southwestern Medical Center, Dallas, TX 75390, USA; 16 Karsh Division of Gastroenterology and Hepatology, Comprehensive Transplant Center, and Samuel Oschin Comprehensive Cancer Institute, Cedars-Sinai Medical Center, Los Angeles, CA 90048, USA

**Keywords:** cholangiocarcinoma, liver disease, oncology, pediatrics, public health

## Abstract

**Background/Objectives**: Early-onset cancer is an emerging global health concern, including in the United States. However, data on early-onset liver and intrahepatic bile duct cancer remain limited. This study aims to fill this gap by analyzing trends in early-onset liver and intrahepatic bile duct cancer in the United States over the past two decades. **Methods**: This study used National Childhood Cancer Registry data to examine temporal trends in early-onset liver and intrahepatic bile duct cancer in the United States. The analysis involved estimating age-adjusted incidence rates of early-onset liver and intrahepatic bile duct cancer, stratified by histological type, ethnicity, and sex. **Results:** In 2021, the age-adjusted incidence rate of early-onset liver and intrahepatic bile duct cancer was estimated at 0.53 per 100,000 population (95% Confidence Interval [CI]: 0.48–0.59). From 2001 to 2021, the age-adjusted incidence rate showed a significant annual percent change (APC) of 1.35% (95% CI: 0.87–1.83%). When stratified by sex, the age-adjusted incidence rate in females increased significantly (APC: 3.07%, 95% CI: 2.26–3.87%) while remaining stable in males. Among racial and ethnic groups, non-Hispanic American Indian and Alaska Native (AIAN) individuals had the highest age-adjusted incidence rate, recorded at 2.67 per 100,000 population (95% CI: 0.95–5.85). By histological type, hepatic carcinoma had the highest age-adjusted incidence rate, significantly increasing over time (APC: 1.47%, 95% CI: 0.96–1.99%). In contrast, the incidence rates for hepatoblastoma and unspecified hepatic tumors remained stable between 2001 and 2021. **Conclusions**: Our study identified an increasing incidence of early-onset liver and intrahepatic bile duct cancer in the United States, primarily driven by cases in females and hepatic carcinoma.

## Simple Summary

In 2021, early-onset liver and bile duct cancer affected about 0.53 people per 100,000 in the United States. Over the past 20 years, cases have been slowly rising by about 1.35% per year. The increase was more noticeable in women, while rates in men stayed the same. Among racial groups, non-Hispanic American Indian and Alaska Native individuals had the highest rates. The most common type, hepatic carcinoma, also increased over time, while other liver tumors remained stable. These findings suggest a growing concern, especially for women and certain racial groups, highlighting the need for more awareness and early detection efforts.

## 1. Introduction

In 2024, an estimated 2 million new cancer cases and 611,720 cancer-related deaths are expected to occur in the United States [1]. While cancer has traditionally been considered a disease primarily affecting older adults, recent epidemiological data reveal a sharp rise in cancer incidence among individuals under 50, known as early-onset cancer [2]. This subset of cancer is often characterized by a more aggressive disease course, subtler initial symptoms, and poorer prognosis compared to cancers diagnosed at older ages [3,4].

According to the Global Burden of Disease Study, the incidence of early-onset cancer has risen by 79%, with associated mortality increasing by 28% over the past three decades [5]. Interestingly, this study found that early-onset liver cancer exhibited the most significant overall decline among all cancer types [5]. However, when stratified by etiology, early-onset liver cancer associated with steatotic liver disease has shown an increasing trend [6]. Given the rising prevalence of steatotic liver disease, along with obesity, among adolescents and young adults, the incidence of early-onset liver cancer is likely to evolve further in the coming years [7,8,9]. Moreover, the epidemiological trends for certain histological subtypes, such as hepatoblastoma, remain understudied, limiting our understanding of their trends and risk factors [10,11,12].

Given these concerns, numerous studies have examined the epidemiological trends in various types of early-onset cancer [13,14,15,16]. However, data on the incidence and burden of early-onset liver and intrahepatic bile duct cancer among young adults in the United States remain limited. Further research is essential to understand the disease dynamics in this population better. The National Childhood Cancer Registry (NCCR) serves as a valuable resource for investigating the epidemiological patterns in early-onset liver and intrahepatic bile duct cancer, offering greater histological detail and broader population coverage compared to the Surveillance, Epidemiology, and End Results (SEER) program (78% in NCCR versus 48% in SEER) [17]. Thus, this study examines trends in early-onset liver and intrahepatic bile duct cancer across the United States from 2001 to 2021, primarily using data from the United States Cancer Statistics via the NCCR, complemented by findings from the Global Burden of Disease Study to characterize temporal patterns and identify potential areas for further research into this growing public health issue.

## 2. Materials and Methods

### 2.1. Data Source

This study analyzed data on early-onset liver and intrahepatic bile duct cancer, defined as liver and intrahepatic bile duct cancer occurring in individuals aged 15–39 years, focusing on age-adjusted incidence rates from 2001 to 2021. The total cancer cases reported in NCCR*Explorer comprised more than 1.8 million cases. The data were sourced from the NCCR, a database maintained by the National Cancer Institute (NCI). The NCCR serves as a robust and rapidly expanding public health surveillance resource to improve the understanding of cancer causes, outcomes, treatments, and long-term effects in children, adolescents, and young adults across the United States [17].

The data were accessed through the NCCR*Explorer, an NCI-managed tool. This publicly available resource, located at https://seer.cancer.gov/statistics-network/ (accessed on 10 February 2025), provides age-adjusted incidence rates for various cancers affecting young adults, including liver and intrahepatic bile duct cancer [18]. NCCR*Explorer does not require a data use agreement, as explicitly stated on its official website. Additionally, it does not necessitate Institutional Review Board approval, given that it does not provide individually identifiable data. This ensures that researchers can access and analyze the dataset without ethical concerns related to patient confidentiality [17].

### 2.2. Estimation Methods

The NCCR*Explorer includes data from 28 NCCR registries, covering approximately 75% of the U.S. population. It records cancer sites based on the International Classification of Childhood Cancer (ICCC) Record Third Edition International Classification of Disease (ICD)-O-3/ International Agency for Research on Cancer 2017. This study specifically examined early-onset liver and intrahepatic bile duct cancer, encompassing hepatoblastoma (ICD-O-3 behavioral code 3, primary site 000-809, and histological types 8970 and 8975), hepatic carcinoma (**1**. ICD-O-3 behavioral code 3, primary site 000-809, and histological types 8160–8162 and 8170–8180 and **2.** ICD-O-3 behavioral code 3, primary site 220–221, and histological types 8010–8041, 8050–8075, 8082, 8120–8122, 8140, 8141, 8143, 8148, 8155, 8158, 8190–8201, 8202, 8210, 8211, 8230, 8231, 8240, 8241, 8244–8246, 8260–8264, 8310, 8320, 8323, 8401, 8430, 8440, 8470, 8480–8490, 8503, 8504, 8510, 8550, 8560–8562, 8570–8573, 9013 and 8170–8180), and unspecified malignant hepatic tumors (ICD-O-3 behavioral code 3, primary site C220-221, and histological types 8000–8005). The detailed histological identification of ICD-O-3 is listed in the NCCR*Explorer database [19]. The age-adjusted incidence rate data were obtained from the NCCR*Explorer database [17], in which values are reported per one million population. To facilitate comparisons with other studies that predominantly use rates per 100,000 population, we converted the original data by multiplying the values by ten [20,21,22,23]. In addition, we also compared these trends with the data from the GBD 2021, using the early-onset liver cancer data to compare with finding from the NCCR database [24]. This comparison was conducted to validate the observed trends across independent, complementary sources and to assess the consistency of incidence estimates

The NCCR was chosen because it provides detailed liver and intrahepatic bile duct cancer data, including histology and race/ethnicity, which are not available in other databases, such as the Global Burden of Disease Study [24]. Demographic information, including sex and race/ethnicity, was collected from 2001 to 2021. Race/ethnicity was categorized as non-Hispanic American Indian and Alaska Native (AIAN), non-Hispanic Asian Pacific Islander (API), non-Hispanic White, non-Hispanic Black, and Hispanic

### 2.3. Statistical Analysis

To account for data variability and uncertainty in statistical modeling, each age-adjusted incidence rate estimate in the study was presented with a corresponding 95% confidence interval (CI). Additionally, the annual percent change (APC) in age-adjusted incidence rates from 2001 to 2021 was calculated, along with 95% CIs, to evaluate temporal trends. An APC with a positive value and *p* < 0.05 was interpreted as an upward trend, while a negative APC with *p* < 0.05 indicated a downward trend. Changes with *p* ≥ 0.05 were considered statistically insignificant. The analysis was conducted using the Joinpoint Regression Program (version 4.9.1.0) developed by the National Cancer Institute.

## 3. Results

### 3.1. Incidence of Early-Onset Liver and Intrahepatic Bile Duct Cancer in the United States

**Figure 1:**
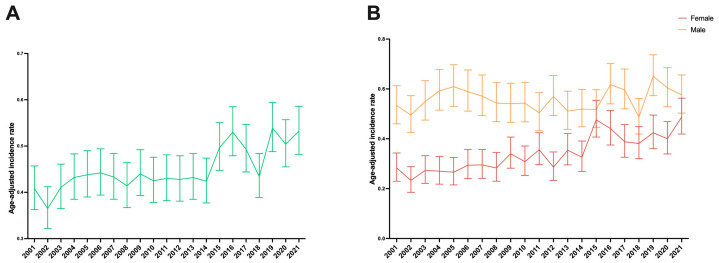
Age-adjusted incidence rate per 100,000 of early-onset liver and intrahepatic bile duct cancer from 2001 to 2021 in the United States (**A**) in both sexes and (**B**) in females and males.

In 2021, the rates of early-onset liver and intrahepatic bile duct cancer age-adjusted incidence were estimated to be 0.53 (95% CI 0.48 to 0.59) per 100,000 population (Table 1 and Figure 1A). From 2001 to 2021, the age-adjusted incidence rate increased by an APC of 1.35 (95% CI 0.87 to 1.83) (Table 1).

### 3.2. Incidence of Early-Onset Liver and Intrahepatic Bile Duct Cancer in the United States, by Sex

In 2021, the age-adjusted incidence rate of early-onset liver and intrahepatic bile duct cancer was slightly higher in males than in females, at 0.58 (95% CI 0.50 to 0.66) and 0.49 (95% CI 0.42 to 0.56) per 100,000 population, respectively (Table 1 and Figure 1B). From 2001 to 2021, the age-adjusted incidence rate of early-onset liver and intrahepatic bile duct cancer increased in females (APC: 3.07%, 95% CI 2.26 to 3.87%) but remained stable in males (Table 1).

### 3.3. Incidence of Early-Onset Liver and Intrahepatic Bile Duct Cancer in the United States, by Race/Ethnicity

**Figure 2:**
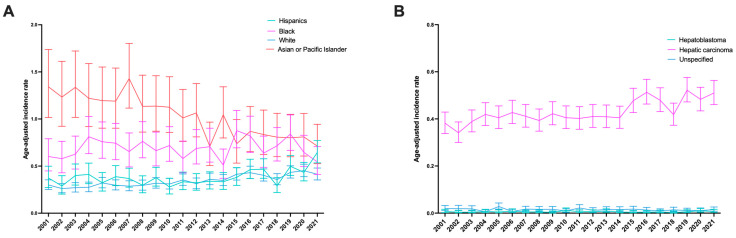
Age-adjusted incidence rate per 100,000 of early-onset liver and intrahepatic bile duct cancer from 2001 to 2021 in the United States (**A**) by ethnicity and (**B**) by histological type.

In 2021, the highest age-adjusted incidence rate per 100,000 population was observed in non-Hispanic AIAN, with an age-adjusted incidence rate of 2.67 (95% CI 0.95 to 5.85) per 100,000 population. This is followed by non-Hispanics API (age-adjusted incidence rate: 0.71, 95% CI 0.53 to 0.94), Hispanics (age-adjusted incidence rate: 0.65, 95% CI 0.53 to 0.77), non-Hispanics black (age-adjusted incidence rate: 0.54, 95% CI 0.41 to 0.71), and non-Hispanics white (0.41, 95% CI 0.35 to 0.48) (Table 1 and Figure 2A). Between 2001 and 2021, the age-adjusted incidence rate increased in non-Hispanic whites (APC: 2.53%, 95% CI 1.96 to 3.10%) and Hispanics (APC: 2.11%, 95% CI 0.64 to 3.60%). However, the age-adjusted incidence rate decreased in API (−3.14%, 95% CI −3.89 to −2.38%) and remained stable in non-Hispanic blacks (Table 1). The APC of age-adjusted incidence rates in non-Hispanic AIANs cannot be analyzed due to incomplete data from the years 2001 to 2021.

### 3.4. Incidence of Early-Onset Liver and Intrahepatic Bile Duct Cancer in the United States, by Histological Type

The highest age-adjusted incidence rate per 100,000 population was observed in hepatic carcinoma, with an age-adjusted incidence rate of 0.51 (95% CI 0.46 to 0.56) per 100,000 population. This is followed by unspecified hepatic tumor and hepatoblastoma, with an age-adjusted incidence rate of 0.02 (95% CI 0.01 to 0.03) and 0.01 (95% CI 0.00 to 0.02) per 100,000 population, respectively (Table 1 and Figure 2B). Between 2001 and 2021, the age-adjusted incidence rate increased for hepatic carcinoma (APC: 1.47%, 95% CI 0.96 to 1.99%) and remained stable for hepatoblastoma and unspecified hepatic tumors (Table 1).

### 3.5. Incidence of Early-Onset Liver and Intrahepatic Bile Duct Cancer in the United States Compared to the Global Burden of Disease Study 2021

In 2021, the age-adjusted incidence rate of early-onset liver and intrahepatic bile duct cancer was estimated at 0.53 (95% CI 0.48 to 0.59) per 100,000 population according to the NCCR database, while the GBD database reported a slightly lower estimate of 0.50 (95% CI 0.48 to 0.53) per 100,000 (Table 2). Between 2001 and 2021, the incidence rate based on NCCR data rose with an annual percent change (APC) of 1.35 (95% CI 0.87 to 1.83), closely aligning with the GBD 2021 estimate of a 1.35 (95% CI 1.16 to 1.55) annual increase (Table 2).

## 4. Discussion

### 4.1. Main Findings

Our analysis revealed a rising incidence of early-onset liver and intrahepatic bile duct cancer, increasing from 0.41 in 2001 to 0.53 per 100,000 population in 2021. This upward trend was further supported by complementary data from the Global Burden of Disease Study 2021. Among racial and ethnic groups, AIAN had the highest age-adjusted incidence rate in 2021. Notably, the increase in early-onset liver and intrahepatic bile duct cancer was primarily driven by females, Hispanics, and non-Hispanic Whites. At the same time, the change in histological subtypes is driven by hepatic carcinoma, which comprises most of the liver and intrahepatic bile duct cancers. The strength of this study lies in utilizing the most up-to-date database, covering three-fourths of the U.S. population, enabling us to examine early-onset liver and intrahepatic bile duct cancer, a relatively understudied gastrointestinal cancer [25,26].

### 4.2. Findings in the Context of Current Literature

These findings build on previous research that examined early-onset liver cancer trends using other databases, such as GBD and SEER [6,27]. Our study’s results are consistent with GBD 2019, which also reported an increasing incidence rate for early-onset liver cancer both globally and in the United States [6]. However, this differs from prior findings based on SEER data [27], which may be attributed to differences in population coverage (75% in NCCR compared to 48% in SEER), differences in histological subtypes (multiple types of histological subtypes in NCCR and hepatocellular carcinoma for Rich et al.) and the study periods (Rich et al. analyzed data from 1992 to 2015, whereas our study spans 2001 to 2021) [27]. Regarding sex, females exhibited increasing incidence rates, but males remained stable over the past two decades. The rising trend could be linked to improved diagnostic practices and the growing prevalence of risk factors, such as an increase in alcohol-associated liver disease in females, as demonstrated in the Rochester database and the National Health and Nutrition Examination Survey [28,29]. In addition, the rising prevalence of metabolic syndrome and metabolic dysfunction-associated steatotic liver disease (MASLD) in adolescents and young adults, which is the etiology of liver cancer, likely contributes to this trend [7,9,30,31]. Beyond traditional risk factors for liver cancer, early-life medication exposure may also play a role [32,33,34]. For instance, a prior multigenerational cohort study found that offspring exposed to antihistamines had a higher risk of developing hepatocellular carcinoma compared to those who were not exposed [35]. In addition, tobacco product use has emerged as a significant contributor to liver cancer [36]. Studies indicate that smokers have a markedly higher risk of developing hepatocellular carcinoma compared to non-smokers [37]. This risk is further exacerbated in individuals with MASLD [38].

Our study highlights the increasing incidence of hepatic carcinoma. However, liver cancer comprises over 20 histological subtypes, such as adenocarcinoma and small-cell carcinoma [39,40,41,42]. Further research with a detailed histological breakdown is needed to enhance our understanding of subtype-specific trends.

Our study found that Hispanic and non-Hispanic white people had the highest increase in early-onset liver and intrahepatic bile duct cancer incidence rates, differing from a previous study projecting the highest rates by 2030 among Hispanics and non-Hispanic blacks [43,44]. This discrepancy stems from our focus on early-onset cases, while the prior study examined all age groups. Liver cancer incidence is influenced by individual-level factors, such as cirrhosis or chronic hepatitis B, which are more common in older individuals, and community-level factors, such as racial and ethnic variations in etiology [45]. For instance, the PNPLA3 gene in Hispanics may accelerate liver inflammation from steatotic liver disease, increasing liver cancer risk [46,47,48,49]. The complexity of liver cancer risk varies across ethnic groups due to genetic, metabolic, and community factors [50,51]. However, early-onset liver cancer remains understudied and requires further investigation [52,53].

### 4.3. Implications for Clinical Practice and Future Research

These results underscore the need for heightened clinical vigilance and earlier screening strategies. Raising awareness among healthcare professionals and policymakers is vital, and liver and intrahepatic bile duct cancer investigations should be considered in younger patients when appropriate. Implementing effective public health strategies, such as reducing risk factors such as alcohol consumption, MASLD, and obesity and enhancing screening, alongside robust research efforts, is essential to mitigating the burden of early-onset liver and intrahepatic bile duct cancer [54,55,56]. Additionally, the influence of early-life exposures, including medication use and tobacco consumption, warrants further investigation, as these factors may contribute to the rising incidence of early-onset liver and biliary tract cancer. Clinicians play a critical role in counseling patients on modifiable risk factors and advocating for targeted public health initiatives aimed at prevention [54,57,58]. Given the growing recognition of genetic predispositions, particularly among Hispanic populations, incorporating genetic risk factors into personalized risk stratification could enhance early detection and intervention efforts [59,60,61].

Despite its rarity, early-onset liver and biliary tract cancer are increasingly common, potentially reflecting both a genuine rise in the incidence rate and improved detection by healthcare providers [62,63]. However, significant gaps remain in our understanding of its etiology and histological evolution. Future iterations of the NCCR might consider incorporating data on the underlying causes of liver cancer, along with expanding coverage beyond the biliary tract, to provide a more detailed analysis of histological trends over time [64]. Additionally, identifying the key drivers of this malignancy is essential for pathologists, gastroenterologists, and researchers to refine diagnostic criteria, enhance surveillance strategies, and improve outcomes for affected patients.

### 4.4. Limitations

The study is limited by the absence of information on the etiology of liver cancer [22,65]. Furthermore, the NCCR dataset does not include data stratified by histological subtypes, such as hepatocellular carcinoma, angiosarcoma, or cholangiocarcinoma, underscoring the need for future research to evaluate the burden of these specific subtypes [42,66]. State-level or county-level data are not available in NCCR*Explorer. Additionally, demographic and socioeconomic variables, including employment, health insurance status, household income, marital status, and education, were not accounted for in this study [67,68]. Although the NCCR encompasses a broader dataset compared to SEER, its coverage remains incomplete, as several states—including Minnesota, North Dakota, South Dakota, Nebraska, Kansas, Wyoming, Arizona, Mississippi, and Alabama—are not represented [17]. Expanding data collection to include these states in future cycles would provide a more comprehensive understanding of childhood liver cancer epidemiology and regional variations [17]. Additionally, the observed differences in trends between hepatic carcinoma and hepatoblastoma should be interpreted with caution. Prior to the establishment of a global histological classification consensus by the Children’s Hepatic Tumors International Collaboration (CHIC) initiative, substantial variability existed in liver cancer classification [69]. Moreover, in some cases, patients received treatment before undergoing a biopsy, leading to potential discrepancies between initial diagnostic biopsy findings and post-treatment resection specimens, which may have significant biological, therapeutic, and prognostic implications [69,70,71]. This highlights the critical need for more detailed histological data to enhance diagnostic precision and improve patient management [66]. Furthermore, the NCCR*Explorer currently lacks epidemiological data on other hepatobiliary tract malignancies, such as extrahepatic biliary tract and gallbladder cancers [72]. Incorporating these cancers into future data cycles would provide valuable insights for policymakers, facilitating more informed decisions regarding cancer prevention, resource allocation, and public health interventions.

## 5. Conclusions

The incidence of early-onset liver and intrahepatic bile duct cancers has exhibited a notable upward trend over the past two decades, largely driven by the increasing incidence rate of hepatic carcinoma. Among racial and ethnic groups, non-Hispanic AIAN individuals had the highest documented incidence rate. However, a significant rise has also been observed among Hispanic and non-Hispanic white populations, suggesting evolving epidemiological patterns. These findings underscore the need for a more tailored approach to liver cancer screening and early detection, particularly in high-risk groups [73,74]. Additionally, the increasing burden of early-onset liver cancer highlights the necessity for proactive prevention strategies, including lifestyle interventions, targeted public health initiatives, and improved access to liver disease surveillance [75,76]. Understanding the underlying factors contributing to these disparities—ranging from genetic predispositions to environmental and metabolic risk factors—will be essential for developing evidence-based policies and clinical guidelines aimed at mitigating the growing impact of early-onset liver and intrahepatic bile duct cancers [77,78,79].

## Figures and Tables

**Table 1:** Age-adjusted incidence rate of early-onset liver cancer in 2001 and 2021, and change from 2001 to 2021.

	2001 Age-Adjusted Incidence Rate per 100,000 Population (95% CI)	2021 Age-Adjusted Incidence Rate per 100,000 Population (95% CI)	2001 to 2021 Annual Percent Change (95% CI)	*p*
Overall	0.41 (0.36 to 0.46)	0.53 (0.48 to 0.59)	1.35 (0.87 to 1.83)	<0.001
By Sex				
Female	0.28 (0.23 to 0.34)	0.49 (0.42 to 0.56)	3.07 (2.26 to 3.87)	<0.001
Male	0.53 (0.46 to 0.61)	0.58 (0.50 to 0.66)	0.32 (−0.27 to 0.92)	0.275
By Race/Ethnicity				
Hispanic	0.37 (0.27 to 0.50)	0.65 (0.53 to 0.77)	2.11 (0.64 to 3.6)	0.007
non-Hispanic Black	0.60 (0.45 to 0.79)	0.54 (0.41 to 0.71)	0.2 (−0.93 to 1.34)	0.719
non-Hispanic White	0.30 (0.25 to 0.35)	0.41 (0.35 to 0.48)	2.53 (1.96 to 3.10)	<0.001
non-Hispanic Asian and Pacific Islander	1.34 (1.02 to 1.74)	0.71 (0.53 to 0.94)	−3.14 (−3.89 to −2.38)	<0.001
non-Hispanic American Indian/Alaska Native	0.53 (0.01 to 2.73)	2.67 (0.95 to 5.85)	N/A	N/A
By Histological type				
Hepatoblastoma	0.01 (0.00 to 0.02)	0.01 (0.00 to 0.02)	0.67 (−2.32 to 3.75)	0.649
Hepatic carcinoma	0.38 (0.34 to 0.43)	0.51 (0.46 to 0.56)	1.47 (0.96 to 1.99)	<0.001
Unspecified hepatic tumor	0.02 (0.01 to 0.03)	0.02 (0.01 to 0.03)	−2.24 (−4.48 to 0.07)	0.056

Abbreviation: CI: confidence interval.

**Table 2:** Age-adjusted incidence rate of early-onset liver cancer in 2021 and change from 2001 to 2021.

	Age-Adjusted Incidence Rate (95% CI)	2001 to 2021 Annual Percent Change (95% CI)	*p*
NCCR	0.53 (0.48 to 0.59)	1.35 (0.87 to 1.83)	*p* < 0.001
GBD 2021	0.50 (0.48 to 0.53)	1.35 (1.16 to 1.55)	*p* < 0.001

Abbreviation: CI: confidence interval; GBD: Global Burden of Disease Study; NCCR: National Childhood Cancer Registry.

## Data Availability

The National Childhood Cancer Registry (NCCR) data can be accessed through the NCCR*Explorer, maintained by the National Cancer Institute (NCI). The data are publicly available at https://seer.cancer.gov/statistics-network/ (accessed on 10 February 2025). Data from the Global Burden of Disease (GBD) study 2021 can be accessed using the Global Health Data Exchange (GHDx) query tool (http://ghdx.healthdata.org/gbd-results-tool, accessed on 10 February 2025) from the Institute for Health Metrics and Evaluation.
